# *β*-glucan Salecan Improves Exercise Performance and Displays Anti-Fatigue Effects through Regulating Energy Metabolism and Oxidative Stress in Mice

**DOI:** 10.3390/nu10070858

**Published:** 2018-07-03

**Authors:** Xi Xu, Yijian Ding, Yunxia Yang, Yan Gao, Qi Sun, Junhao Liu, Xiao Yang, Junsong Wang, Jianfa Zhang

**Affiliations:** 1 Center for Molecular Metabolism, Nanjing University of Science & Technology, Nanjing 210094, China; xuxi@njust.edu.cn (X.X.); yyx0625@outlook.com (Y.Y.); 113102000225@njust.edu.cn (Y.G.); 311020270@njust.edu.cn (Q.S.); craigdragon@icloud.com (J.L.); yangxiaodirk@163.com (X.Y.); wang.junsong@gmail.com (J.W.); 2 Department of Physical Education, Nanjing University of Science & Technology, Nanjing 210094, China; felao@163.com

**Keywords:** *β*-glucan, Salecan, exercise-induced fatigue, biomarker, oxidative stress

## Abstract

Fatigue induced by prolonged exercise not only leads to the decrease of exercise capacity, but also might be the cause of many diseases. In consideration of the side effects of pharmacological drugs, dietary supplements seem to be a better choice to ameliorate exercise-induced fatigue. The present study aimed to investigate the anti-fatigue effect of Salecan, a novel water-soluble *β*-glucan, during exercise and explore the underlying mechanisms. Male Institute of Cancer Research (ICR) mice were divided into five groups, including the Rest group and the other four Swim-groups treated with Salecan at 0, 25, 50, and 100 mg/kg/day for four weeks. Salecan treatment markedly increased the exhaustive swimming time of mice in the forced swimming test. Exercise fatigue and injury-related biochemical biomarkers including lactate, blood urea nitrogen (BUN), creatinine kinase (CK), alanine transaminase (ALT), and aspartate transaminase (AST) were ameliorated by Salecan. Salecan reversed the decreased serum glucose levels and glycogen contents caused by exercise. In addition, Salecan improved oxidative stress induced by exercise through regulating Nrf2/HO–1/Trx signaling pathway. Thus, the beneficial effects of Salecan against fatigue may be due to its positive effects on energy metabolism and antioxidation defence. Our results suggest that Salecan could be a novel potential candidate for anti-fatigue dietary supplements.

## 1. Introduction

Fatigue, a common and complicated symptom, could have negative effects on health conditions, work efficiency, life quality, and social relationships. According to the causes of fatigue, it can be classified as physical, mental, or disease-related [1,2,3,4,5]. Exercise-induced fatigue is not fully understood, although it has been investigated by plenty of researchers. The mechanisms that contribute to exercise-induced fatigue may include the decrease of energy stores, the accumulation of the end products of metabolism, disorder of the internal environment, and high levels of oxidative stress [6,7,8,9]. The purposes of anti-fatigue studies are to increase exercise capacity and to shorten the recovery time after fatigue. In consideration of the side effects of pharmacological drugs, such as safety and legality issues, dietary supplements seem to be a better choice to treat exercise-induced fatigue.

Because no single biomarker could precisely reflect the extent of exercise-induced fatigue, combinations of different biomarkers have been widely used [10]. According to the test methods, biomarkers of exercise-induced fatigue could be classified as dry, wet, or volatile [10]. Dry biomarkers are usually least invasive and easy to detect. Power output measures, electrophysiological measures, and cardiologic measures are the most commonly used dry biomarkers [10]. Most of the wet biomarkers derive from blood and only some from the saliva, urine, or tissue [10]. The most widely used wet biomarkers are those originating from muscle damage, adenosine triphosphate (ATP) depletion, oxidative stress, or immunological compromise [10,11,12]. Because oxygen delivery and utilization are important to sustain the endurance performance of muscle, maximal oxygen uptake is considered as one of the volatile biomarkers [10].

**Figure 1:**
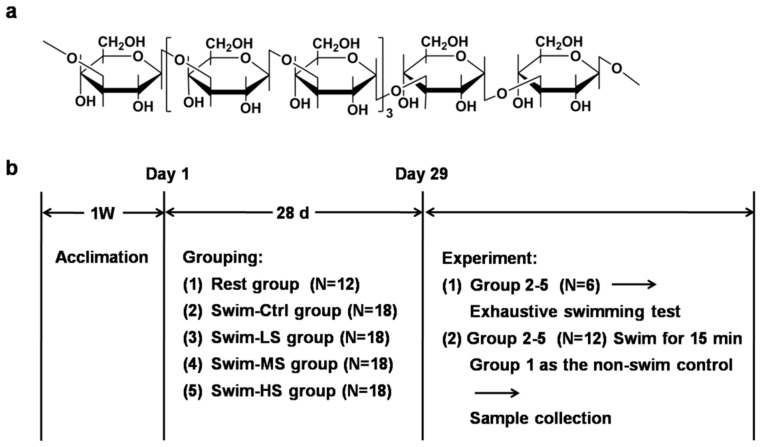
Chemical structure of the repeated unit of Salecan (**a**) and the experiment scheme (**b**). Rest group: mice that received phosphate buffer saline (PBS) intragastrically and did not take the swimming test; Swim-Ctrl, Swim-LS, Swim-MS, and Swim-HS groups: mice that received 0, 25, 50, and 100 mg/kg of Salecan intragastrically respectively for four weeks before taking a 15-min swimming test.

*β*-glucans are naturally occurring polysaccharides with poly-branched *β*-(1-3)-d-glucans or *β*-(1-6)-d-glucose side chains, and are widely distributed in the cell walls of fungi or bacteria, cereal plants, and seaweeds [13,14]. A number of studies have reported the biological functions of *β*-glucans, such as modulating the immune system, protecting against tumors, and reducing the absorption of cholesterol and fat [15,16]. Salecan is a novel water-soluble *β*-glucan produced by Agrobacterium sp. ZX09. It is an extracellular polysaccharide with a structure consisting of the following repeating unit: 3)-*β*-d-Glcp-(1→3)-[*β*-d-Glcp-(1→3)-*β*-d-Glcp-(1→3)]3-*α*-d-Glcp-(1→3)-*α*-d-Glcp-(1→ (Figure 1a) [17]. The safety of Salecan has been examined in a previous acute and subchronic experiment [18]. Previous studies also found that Salecan exhibits multiple biological activities, such as protecting against liver injury [13,19,20] and gastrointestinal diseases [21,22]. Because previous studies suggested the possible anti-oxidation ability of Salecan [13,19], we asked whether Salecan could be a dietary supplement to ameliorate exercise-induced fatigue. In the present study, we used a mice model to simulate exercise-induced fatigue, and examined the anti-fatigue effects of Salecan during exercise.

## 2. Materials and Methods

### 2.1. Preparation of Salecan

Salecan was extracted from the fermentation broth of Agrobacterium sp. ZX09 by centrifugation and ethanol precipitation, and was further purified as previously described [17,23,24]. Briefly, gel filtration chromatography was performed with a Sepharose CL-4B (Pharmacia, Shanghai, China) column (1.5 × 60 cm), and the polysaccharides were eluted with 50 mmol/L phosphate buffer, pH 7.2, at the rate of 1 mL/min. Fractions containing polysaccharides were collected, and the purity of purified Salecan was determined to be more than 95%.

### 2.2. Mice and Treatment

Male six-weeks-old Institute of Cancer Research (ICR) mice, purchased from the Model Animal Research Center of Nanjing University (Nanjing, China), were used in the study. The animals were maintained under a 12/12 h light/dark cycle with lights on at 7:00 a.m. and off at 7:00 p.m., and with free access to regular chow food and water. The mice were randomly divided into five groups: (1) Rest group that received phosphate buffer saline (PBS) intragastrically (*N* = 12); (2) to (5) Swim-Ctrl, Swim-LS, Swim-MS, and Swim-HS groups that received 0, 25, 50, and 100 mg/kg of Salecan intragastrically, respectively (*N* = 18 in each group). After treatment with PBS or Salecan once a day for 28 consecutive days, six mice in group (2) to (5) were assigned to take the forced swimming test, while the 12 mice left in group (2) to (5) underwent a 15-min swimming test, and the mice in group (1) were used as the non-swim control (Figure 1b). The body weights of mice were recorded weekly. Sample size was determined by power analysis with the following assumptions: α of 0.05 (two-tailed), power of 90%. The values used in the calculation were based on the preliminary data obtained in our laboratory, as well as on the published studies that used these biochemical techniques. All animal care and use procedures were approved by the Institutional Animal Care and Use Committee at the Nanjing University of Science and Technology (ACUC-NUST-20170026), and were performed according to the Chinese guidelines for the care and use of laboratory animals.

### 2.3. Forced Swimming Test

One hour after the last PBS or Salecan treatment, the forced swimming test was performed as previously described with minor modifications [25]. Briefly, mice were placed individually in a swimming pool filled with water (25 ± 2 °C) to a depth of 30 cm. A tin wire (5% of body weight) was attached to the tail root of each mouse. The exhaustive swimming time was recorded when the loss of coordinated movements and failure to return to the surface within 7 s were observed in each mouse.

### 2.4. Biochemical Analysis

One hour after the last PBS or Salecan treatment, the mice in group (2) to (5) were forced to swim in water at 25 ± 2 °C for 15 min without any loads, and the mice in group (1) were used as the non-swim control. After the swimming exercise, all mice were sacrificed, and the blood samples, livers, skeletal muscles, hearts, and kidneys were collected. Serum alanine transaminase (ALT), aspartate transaminase (AST), creatinine kinase (CK), blood urea nitrogen (BUN), glucose levels, and lactate dehydrogenase (LDH) activity were measured using an Olympus AU2700 automatic biochemical analyzer (Olympus, Tokyo, Japan). The concentrations of lactate in the serum were determined according to the instructions (Jiancheng, Nanjing, China).

### 2.5. Tissue Glycogen Examination and Oxidative Stress-Related Parameters Analysis

The livers and muscles were homogenized with saline and the concentrations of glycogen and oxidative stress-related parameters (malondialdehyde (MDA), glutathione (GSH), superoxide dismutase (SOD)) were determined using available kits (Jiancheng, Nanjing, China).

### 2.6. Tissue Energy Metabolic Enzymes Analysis

Mice treated with PBS or Salecan (100 mg/kg) separately for three days were analyzed for the activities of energy metabolic enzymes in the liver and muscle (*N* = 6). The liver or muscle was homogenized and centrifuged. The activities of pyruvate kinase (PK), succinate dehydrogenase (SDH), malate dehydrogenase (MDH), and Na^+^-K^+^-ATPase in the supernatant were analyzed with commercially available kits (Jiancheng, Nanjing, China).

### 2.7. RNA Isolation and Real-Time Polymerase Chain Reaction (PCR) Analysis

Total RNA was extracted using TRIzol (Invitrogen, Carlsbad, CA, USA). Reverse transcript reaction was carried out by commercial reverse transcript enzyme (KeyGene, Nanjing, China). Quantitative real-time PCR was carried out using an ABI 7300 real-time PCR system with a cDNA sample, and amplification was carried out in a 20 μL reaction volume containg 1× SYBR Green PCR Master Mix (Applied Biosystems, Foster City, CA, USA). The primers used were nuclear factor erythroid 2-related factor 2 (Nrf2), F: TCCGCTGCCATCAGTCAGTC, R: ATTGTGCCTTCAGCGTGCTTC; hemeoxygenase-1 (HO-1), F: TGCAGGTGATGCTGACAGAGG, R: GGGATGAGCTAGTGCTGATCTGG; thioredoxin (Trx)1, F: CGTGGTGGACTTCTCTGCTACGTGGTG, R: GGTCGGCATGCATTTGACTTCACAGTC; Trx2, F: GCTAGAGAAGATGGTCGCCAAGCAGCA, R: TCCTCGTCCTTGATCCCCACAAACTTG; thioredoxin reductase (TrxR)1, F: GGCCAACAAAATCGGTGAACACATGGAAG, R: CGCCAGCAACACTGTGTTAAATTCGCCCT; TrxR2, F: GTCCCCTCCCACATCAAAAAACTCCCAAC, R: GGCCCACAGGACAGTGTCAAAGGTGC; glutaredoxin (Grx)1, F: TGCAGAAAGACCCAAGAAATCCTCAGTCA, R: TGGAGATTAGATCACTGCATCCGCCTATG; Grx2, F: CATCCTGCTCTTACTGTTCCATGGCCAA, R: TCATCTTGTGAAGCGCATCTTGAAACTGG; glutathione reductase (GR), F: GCCTTTACCCCGATGTATCACGCTGTG, R: TGTGAATGCCAACCACCTTTTCCTCTTTG. Relative expression in comparison with that of GAPDH was calculated using the comparative computed tomography method.

### 2.8. Western Blot

Cytoplasmic and nuclear proteins in the liver and muscle were extracted using nuclear and cytoplasmic protein extraction kits (Beyotime, Shanghai, China). Proteins were separated by SDS-PAGE (10% gel), transferred to 0.22 μm polyvinylidene fluoride (PVDF) membranes (Biosharp). After blocking with 5% skim milk powder (Biosharp) in TBST (0.1% TWEEN-20), the membranes were incubated with Nrf2 primary antibody (Proteintech, Chicago, IL, USA) overnight, washed with TBST, and incubated with secondary antibody at room temperature for one hour. Chemiluminescent Substrate System from KPL was utilized for final detection.

### 2.9. Statistical Analysis

The statistical analyses of the results were performed using GraphPad Prism 5 software (GraphPad Software, San Diego, CA, USA). The data were first analyzed by one-way ANOVA, and then unpaired or two-tail paired *t*-test was used to evaluate the significance of the differences between two groups when necessary. Spearman rank correlation analyses were performed to evaluate the correlation between different parameters. The following terminology is used to denote the statistical significance: * *p* < 0.05, ** *p* < 0.01, and *** *p* < 0.001.

## 3. Results

### 3.1. Effects of Salecan on the Body Weight and Histopathology of Mice

**Figure 2:**
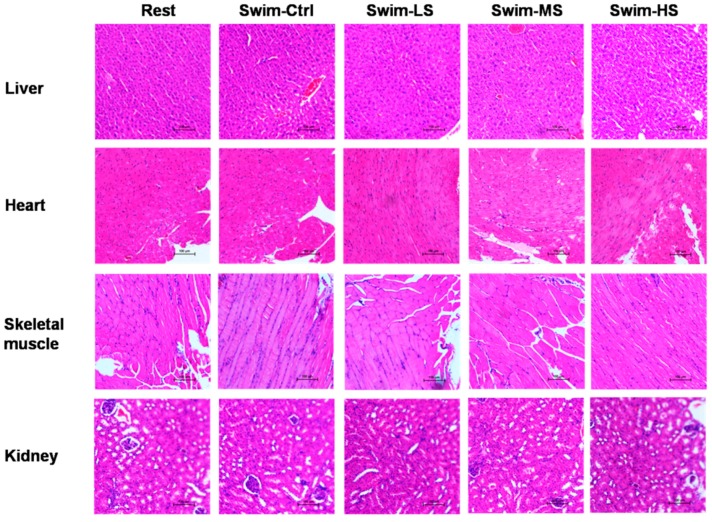
The effects of Salecan on the histopathology of liver, heart, skeletal muscle, and kidney of mice in different groups. Thick sections of 5 μm were stained with hematoxylin-eosin staining (H & E) (magnification, ×200).

The body weights of mice were recorded and the results showed that no significant difference was found among the five groups (data not shown). The histological analyses were also performed to evaluate the effect of Salecan on different tissues after acute exercise for 15 min. Salecan treatment did not significantly change the structure of hepatic sinusoids in liver. Cardiomyocytes in the heart showed no sign of hypertrophy and hyperplasia, and neither did the rhabdomyocytes in the skeletal muscle. The morphology of glomerulus and renal tubules did not differ among the control and Salecan-treated groups (Figure 2). Thus, Salecan treatment did not significantly change the body weight and histopathology of mice.

### 3.2. Salecan Prolonged the Exhaustive Swimming Time in the Forced Swimming Test

**Figure 3:**
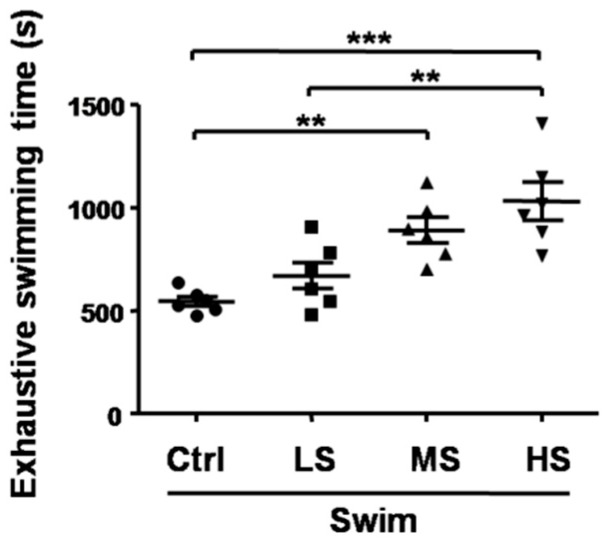
The effects of Salecan on the exhaustive swimming times of mice from four groups. Data are expressed as mean ± SD, *N* = 6, ** *p* < 0.01, *** *p* < 0.001. Swim-Ctrl, the control group; Swim-LS, the low-dose of Salecan-treated group; Swim-MS, the medium-dose of Salecan-treated group; Swim-HS, the high-dose of Salecan-treated group.

The forced swimming test, the most widely used animal model to investigate the anti-depression effect of current or novel molecules, has recently been used to evaluate the anti-fatigue activities of certain agents [26,27]. In the forced swimming test, prolonged swimming time indicates the increase of exercise capacity and decrease of fatigue [27]. Compared with the Swim-Ctrl group (545.2 ± 57.1 s), the forced swimming times in the Salecan-treated groups were longer (Swim-LS: 670.8 ± 157.4 s; Swim-MS: 890.8 ± 150.2 s; Swim-HS: 1031.3 ± 223.8 s), and the differences were statistically significant (*p* < 0.001, the statistical significance of the data between different treatments was shown in the figure) (Figure 3). These results indicated that Salecan had significant anti-fatigue activity and could increase exercise endurance in a dose-dependent way (correlation analysis between Salecan dosage and exhaustive swimming time: *r* = 0.79, *p* < 0.001).

### 3.3. Salecan Ameliorated Exercise Fatigue and Injury-Related Biochemical Parameters after Strenuous Exercise

**Figure 4:**
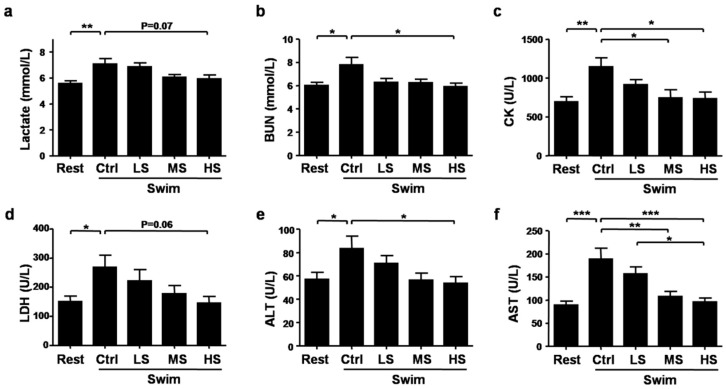
The effects of Salecan on exercise fatigue and injury-related biochemical parameters including (**a**) Lactate, (**b**) blood urea nitrogen (BUN), (**c**) creatinine kinase (CK), (**d**) lactate dehydrogenase (LDH), (**e**) alanine transaminase (ALT), and (**f**) aspartate transaminase (AST). Data are expressed as mean ± SD, N = 12, * *p* < 0.05, ** *p* < 0.01, *** *p* < 0.001. Rest, the control group without swimming; Swim-Ctrl, the control group that swim for 15 min before sample collection; Swim-LS, the low-dose of Salecan-treated group that swim for 15 min before sample collection; Swim-MS, the medium-dose of Salecan-treated group that swim for 15 min before sample collection; Swim-HS, the high-dose of Salecan-treated group that swim for 15 min before sample collection.

Several biochemical parameters have been used to evaluate the extent of muscle fatigue and injury after exercise, such as lactate, BUN, CK, LDH, ALT, and AST [10,11,12,28]. Compared with the Rest group, the concentrations of lactate were higher after exercise by 26.72%, Salecan treatment decreased lactate levels after exercise by 3.40%, 14.55%, and 16.43% respectively (*p* < 0.05, the statistical significance of the data between different treatments was shown in the figure) (Figure 4a). Similar trends were found in the levels of BUN (Swim-Ctrl versus Rest: increased by 29.64%, Swim-LS versus Swim-Ctrl: decreased by 19.66%, Swim-MS versus Swim-Ctrl: decreased by 19.98%, Swim-HS versus Swim-Ctrl: decreased by 24.04%, *p* < 0.01) and CK (Swim-Ctrl versus Rest: increased by 64.88%, Swim-LS versus Swim-Ctrl: decreased by 20.02%; Swim-MS versus Swim-Ctrl: decreased by 35.10%, Swim-HS versus Swim-Ctrl: decreased by 35.72%, *p* < 0.01) (Figure 4b,c). LDH activities were increased after exercise but decreased in the Swim-HS group (*p* = 0.07) (Figure 4d). Strenuous exercise also caused the increase of ALT and AST levels, which were markedly decreased by Salecan treatment (ALT: *p* < 0.05; AST: *p* < 0.001; the statistical significance of the data between different treatments was shown in the figure) (Figure 4e,f). Correlation analyses were performed between each biochemical parameter and Salecan dosage or exhaustive swimming time. The results demonstrated that Salecan dosage was negatively correlated with lactate, BUN, CK, LDH, ALT, and AST levels (Table 1). Exhaustive swimming time was negatively correlated with CK and AST levels (Table 1).

### 3.4. The Regulatory Effect of Salecan on Energy Metabolism

**Figure 5:**
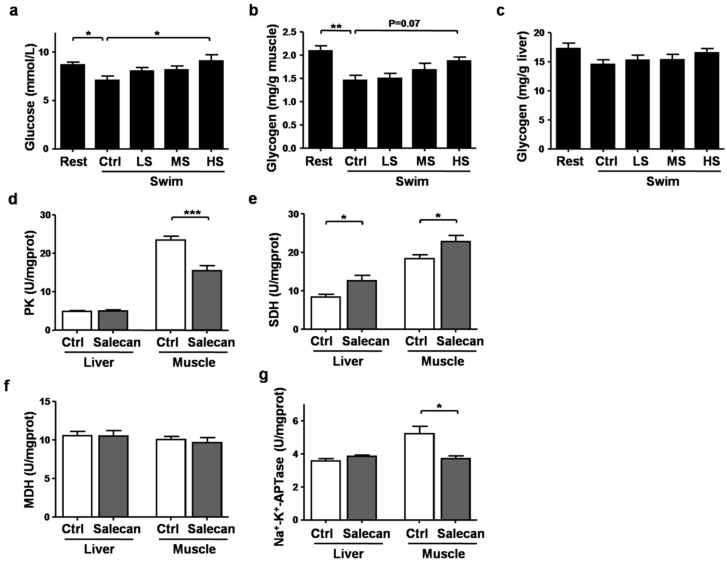
The regulatory effects of Salecan on glucose metabolism including (**a**) serum glucose, (**b**) muscle glycogen content, (**c**) liver glycogen contents and the effects of Salecan on energy metabolic enzymes including (**d**) pyruvate kinase (PK), (**e**) succinate dehydrogenase (SDH), (**f**) malate dehydrogenase (MDH), and (**g**) Na^+^-K^+^-ATPase. Data are expressed as mean ± SD, *N* = 6, * *p* < 0.05, ** *p* < 0.01, *** *p* < 0.001.

Depletion of energy stores could also lead to exercise fatigue, and this could be revealed by the levels of blood glucose and the glycogen contents in the liver and muscle [29]. Compared with the Rest group, serum glucose levels were decreased after exercise in the Swim-Ctrl group by 18.22%, but elevated by 13.28%, 14.83%, and 28.18% in the Swim-LS, Swim-MS, and Swim-HS groups, respectively, however, because of the limited sample numbers, the ANOVA analysis did not reach significance (*p* = 0.06) (Figure 5a). Positive correlation was found between Salecan dosages and glucose levels (Table 1). In accordance with the trend of glucose levels in the serum, the glycogen contents in the muscle were also decreased after exercise by 30.06%, but increased in the Swim-HS group by 28.42% compared with the Swim-Ctrl group (*p* = 0.06) (Figure 5b). The glycogen content in the muscle was also positively correlated with Salecan dosage (Table 1). The glycogen contents of the liver among different treatments did not reach significance (Figure 5c).

To further investigate the effects of Salecan on the main processes of energy metabolism, we compared the activities of key energy metabolic enzymes, including PK, SDH, MDH, and Na^+^-K^+^-ATPase, in mice treated with PBS or Salecan for three days. Salecan significantly decreased the activity of PK in muscle (Figure 5d) and increased the activity of SDH in liver and muscle (Figure 5e). Salecan did not have an effect on MDH levels, but further decreased the activity of Na^+^-K^+^-ATPase in muscle (Figure 5f,g). As CK and AST were two sensitive fatigue and injury-related indicators in response to Salecan treatment, we further performed correlation analyses between these biochemical indicators and energy metabolic enzymes. The data showed that the CK and AST levels are associated with the fall in muscle glycolytic markers, PK and Na^+^-K^+^-ATPase (Table 2). Both muscle and liver SDH levels were negatively correlated with the AST levels (Table 2).

### 3.5. Salecan Alleviated the Oxidative Stress in Mice after Acute Exercise

**Figure 6:**
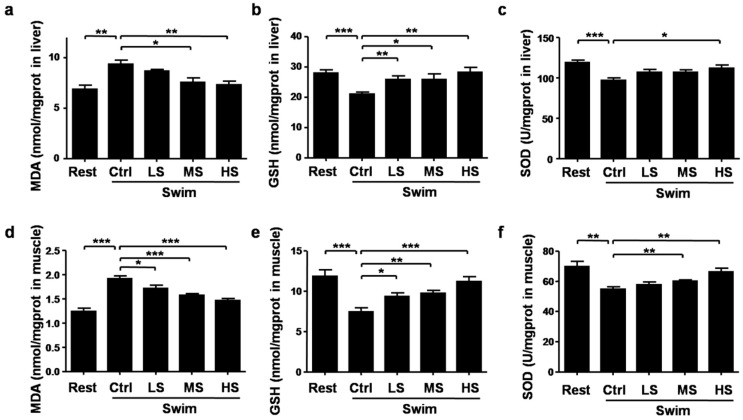
The effects of Salecan on oxidative stress-related parameters in the liver and muscle of mice from different groups. (**a**) Liver MDA; (**b**) liver GSH; (**c**) liver SOD; (**d**) muscle MDA; (**e**) muscle GSH; and (**f**) muscle SOD. Data are expressed as mean ± SD, *N* = 6, * *p* < 0.05, ** *p* < 0.01, *** *p* < 0.001. Rest, the control group without swimming; Swim-Ctrl, the control group that swim for 15 min before sample collection; Swim-LS, the low-dose of Salecan-treated group that swim for 15 min before sample collection; Swim-MS, the medium-dose of Salecan-treated group that swim for 15 min before sample collection; Swim-HS, the high-dose of Salecan-treated group that swim for 15 min before sample collection.

During exercise, oxidative stress could lead to muscle damage and fatigue, thus we investigated the effects of Salecan on oxidative stress. The oxidative stress-related molecules including MDA, GSH, and SOD were examined. Compared with the Rest group, MDA levels in the liver and muscle were significantly increased after strenuous exercise, this increase was partly reversed by Salecan treatment (liver: *p* < 0.001; muscle: *p* < 0.001; the statistical significance of the data between different treatments was shown in the figure) (Figure 6a,d). The GSH contents in the liver and muscle were markedly decreased after exercise, but elevated after Salecan treatment (liver: *p* < 0.01; muscle: *p* < 0.05) (Figure 6b,e). Similar to the changes of the GSH contents, the SOD activities were significantly decreased after exercise, but increased in Salecan-treated groups (liver: *p* < 0.01; muscle: *p* < 0.001) (Figure 6c,f). Salecan dosage was positively correlated with muscle GSH levels, and negatively correlated with muscle MDA levels (Table 1). The levels of MDA (the most sensitive biochemical indicator changed in response to Salecan treatment) were not associated with the changes in energy metabolic enzymes (Table 2).

**Figure 7:**
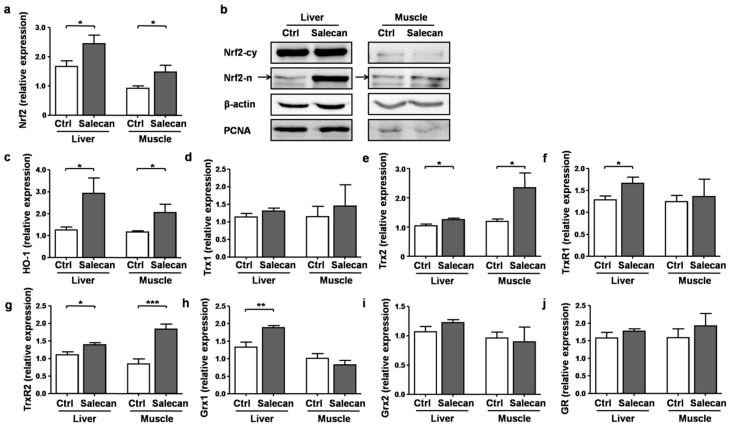
The mRNA and protein levels of (**a**,**b**) Nrf2; mRNA expression levels of (**c**) HO-1, (**d**) Trx1, (**e**) Trx2, (**f**) TrxR1, (**g**) TrxR2, (**h**) Grx1, (**i**) Grx2, and (**j**) GR in the liver and muscle of mice from the control and Salecan-treated groups were analyzed. Ctrl, the control group that received phosphate buffer saline (PBS) for three days; Salecan, the group that received high-dose of Salecan treatment for three days. PCNA, proliferating cell nuclear antigen. Data are expressed as mean ± SD, *N* = 6, * *p* < 0.05, ** *p* < 0.01, *** *p* < 0.001.

To further elucidate the molecular mechanisms of Salecan’s regulatory role on oxidative stress, we performed real-time PCR and Western blot to study the effects of Salecan on the expression patterns of some important antioxidant signaling pathways. Nrf2, a basic region leucine-zipper transcription factor, is one of the key mediators involved in the antioxidant system in vivo [30]. Under oxidative stress, Nrf2 could translocate from the cytoplasm to the nucleus, bind to the antioxidant response element (ARE), and thereby regulate the expression of a large battery of genes involved in the cellular antioxidant defence, such as HO-1, Trx, glutathione S-transferase-α1 (GST-α1), and quinoneoxidoreductasel (NQO1) [30,31,32,33]. The real-time PCR results demonstrated that Salecan significantly increased the expression levels of Nrf2 (Figure 7a). The protein levels of Nrf2 in the cytoplasm were not significantly changed after Salecan treatment, however, the expression levels of nuclear Nrf2 were markedly increased in both liver and muscle (Figure 7b), suggesting the activation of Nrf2 by Salecan. Further analyses of the downstream signaling pathways of Nrf2 demonstrated that Salecan also increased the mRNA levels of HO-1, Trx2, and TrxR2 in both liver and muscle, and only increased the levels of TrxR1 and Grx1 in liver, but did not have a significant effect on the expression of Trx1, Grx2, and GR (Figure 7c–j). Only TrxR2 expression levels were negatively correlated with AST levels (*r* = −0.62, *p* < 0.05). Together, these results suggested that Salecan could regulate oxidative stress through the Nrf2/HO-1/Trx signaling pathway, thus exerting an anti-fatigue effect during exercise.

## 4. Discussion

Searching for effective treatments to alleviate exercise-induced fatigue has been an interesting research project for sports physiologists and nutritionists. In this study, we found that a new type of water-soluble *β*-glucan, Salecan, could markedly alleviate exercise-induced fatigue. Salecan treatment significantly prolonged the exhaustive swimming time of mice in the forced swimming test. Exercise fatigue and injury-related biochemical parameters (lactate, BUN, CK, LDH, ALT, AST) were ameliorated by Salecan. We also demonstrated that Salecan improved exercise fatigue through regulating energy metabolism and Nrf2/HO-1/Trx signaling-related antioxidation defence. Our results suggested that Salecan could be a novel potential candidate for anti-fatigue dietary supplements.

Combinations of a variety of biomarkers have been used to evaluate the extent of exercise-induced fatigue. In this study, both dry and wet biomarkers have been used. The exhaustive swimming time of mice could be considered as one of the dry biomarkers to reflect the extent of exercise endurance and fatigue. Salecan treatment prolonged the time to exhaustion in mice, particularly in the Swim-MS and Swim-HS groups, indicating the anti-fatigue effects of Salecan on mice. Wet biomarkers derived from blood and tissues were also examined. During strenuous exercise, the generation of ATP shifts from aerobic processes to anaerobic glycolysis or glycogenolysis. Lactate is a side product of the anaerobic pathway and the accumulation of lactate often leads to fatigue during exercise [10,11,34]. Lactate levels were significantly increased after exercise but decreased in the Salecan-treated groups. The decreased LDH activities after Salecan treatment might be the feedback of the decreased levels of lactate. Another important biomarker, CK, is one of the indicators of muscle damage [10]. Strenuous exercise that damages skeletal muscle cell structure at the level of sarcolemma and Z-disks results in an increase in CK levels [12,35]. From the results of serum biomarkers, we concluded that Salecan could significantly alleviate exercise fatigue and injury-related biochemical parameters after acute exercise. Except for the biomarkers mentioned above, other biomarkers such as C-terminal agrin fragment, muscle damage parameters (such as myoglobin), and inflammation indicators (including blood leukocytes, interleukine, cortisol, and C-reactive protein) have also been used in recent years [10,11]. Thus, an in-depth examination of these fatigue-related biomarkers need to be done to clarify the anti-fatigue effect of Salecan in future research.

Oxidative stress produced during strenuous exercise may not be responsible only for exercise-induced fatigue, but also for impaired recovery from exercise [36]. Increased reactive oxygen species (ROS) production during strenuous exercise leads to the oxidation of proteins, lipids, or nucleic acids [37]. The production of ROS during exercise is also accompanied with a reduction of antioxidant capacity [8]. Thus, we examined the levels of oxidative stress with indicators including MDA, GSH, and SOD in different groups. The results were consistent in the livers and muscles of Salecan-treated mice, and demonstrated that Salecan significantly reduced oxidative stress during acute exercise. We further studied the molecular mechanisms involved in antioxidation defence. The basic region leucine-zipper transcription factor Nrf2 is a key molecule regulating the cellular antioxidant response. Under oxidative stress, Nrf2 translocates to the nucleus of the cell, where it forms heterodimers with other transcription factors such as c-Jun and small Maf proteins, binds to the ARE, and regulates the expression of downstream genes involved in the cellular antioxidant defense, such as HO-1, Trx, GST-α1, and NQO1 [30,31,32,33]. The real-time PCR analyses revealed that Nrf2 expression was enhanced after Salecan treatment, and the downstream HO-1 and Trx signaling (especially Trx2-TrxR2 signaling) were also activated.

Glycolysis is the major short term energy source, and when it is altered, other changes occur such as the change in energy source. The correlation analyses demonstrated that the Salecan dosage was positively correlated with muscle glycogen, GSH, and glucose levels, and negatively correlated with MDA, AST, CK, lactate, ALT, LDH, and BUN levels (Table 1). Thus, increasing levels of Salecan appeared to be associated with reduction in muscle glycogen usage and increases in muscle GSH and serum glucose levels. These changes also appeared to be associated with the reduction of cellular damage due to the falls in CK and liver enzyme levels. Thus, *β*-Glucan Salecan seems to provide an increased ability to resist the onset of the change from glycolysis to other energy sources and in doing so, delays the onset of oxidative damage. It was anticipated that these would also be associated with an increase in serum insulin levels [38], and this needs to be examined in the future.

Strenuous exercise is a very energy-consuming process, which might result in the imbalance of the internal environment. Fatigue induced by prolonged exercise not only leads to the decrease of exercise capacity, but also might be the cause of many diseases including vascular and heart diseases, and skeletal and muscular system diseases [1,7,39]. Here, we demonstrated that Salecan could significantly alleviate exercise-induced fatigue. The beneficial effects of Salecan against fatigue may be due to its positive effects on energy metabolism and antioxidation capacity. Except for the traditional biochemical biomarkers for exercise-induced fatigue, other metabolites related to fatigue need to be further investigated to better understand the role of Salecan. These findings suggest a potential use for Salecan as an anti-fatigue dietary supplement in exercise or other factors inducing the fatigue symptom.

## Figures and Tables

**Table 1:** Correlation analyses between each biochemical parameter and Salecan or exhaustive swimming time were performed. BUN—blood urea nitrogen; CK—creatinine kinase; LDH; ALT—alanine transaminase; AST—aspartate transaminase; MDA—malondialdehyde; GSH—glutathione.

Correlation Coefficient	Salecan	Exhaustive Swimming Time
Lactate	−0.41 **	−0.23
BUN	−0.32 *	−0.10
CK	−0.44 **	−0.49 *
LDH	−0.33 *	−0.13
ALT	−0.40 **	−0.32
AST	−0.59 ***	−0.44 *
Glucose	0.49 **	0.49 *
Muscle glycogen	0.52 **	0.48 *
Muscle MDA	−0.71 ***	−0.46 *
Muscle GSH	0.52 **	0.41 *

* *p* < 0.05, ** *p* < 0.01, and *** *p* < 0.001.

**Table 2:** Correlation analyses between biochemical parameters (creatinine kinase (CK), aspartate transaminase (AST), and malondialdehyde (MDA)) and energy metabolic enzymes. PK—pyruvate kinase; SDH—succinate dehydrogenase.

Correlation Coefficient	CK	AST	MDA
PK-muscle	0.58 *	0.73 **	0.27
SDH-liver	−0.50	−0.65 *	−0.48
SDH-muscle	−0.47	−0.61 *	−0.53
Na^+^-K^+^-ATPase-muscle	0.49	0.66 *	0.00

* *p* < 0.05 and ** *p* < 0.01.
